# Punicalagin Inhibits African Swine Fever Virus Replication by Targeting Early Viral Stages and Modulating Inflammatory Pathways

**DOI:** 10.3390/vetsci11090440

**Published:** 2024-09-19

**Authors:** Renhao Geng, Dan Yin, Yingnan Liu, Hui Lv, Xiaoyu Zhou, Chunhui Bao, Lang Gong, Hongxia Shao, Kun Qian, Hongjun Chen, Aijian Qin

**Affiliations:** 1 College of Veterinary Medicine, Yangzhou University, Yangzhou 225009, China; gengrenhao57@gmail.com (R.G.); ydan0203@126.com (D.Y.); hui.lv7@outlook.com (H.L.); zxiaoyu95@163.com (X.Z.); eva.bao@genscriptprobio.com (C.B.); hxshao@yzu.edu.cn (H.S.); 2 Jiangsu Co-Innovation Center for Prevention and Control of Important Animal Infectious Diseases and Zoonoses, Yangzhou University, Yangzhou 225009, China; 3 Shanghai Veterinary Research Institute, Chinese Academy of Agricultural Sciences (CAAS), Shanghai 200241, China; liuyingnan@shvri.ac.cn; 4 College of Veterinary Medicine, South China Agricultural University, Guangzhou 510640, China; gonglang@scau.edu.cn

**Keywords:** African swine fever virus, library screen, punicalagin, replication stages, NF-κB/STAT3/NLRP3

## Abstract

**Simple Summary:**

The spread of the African swine fever virus (ASFV) poses a great threat to the global pig industry and new measures for prevention and control are urgently needed. With no effective vaccines, drug development against ASFV is crucial. This study screened 536 antiviral compounds and identified that punicalagin, from pomegranate peel, significantly inhibited ASFV replication in various cell lines in vitro. Punicalagin acted on early viral replication stages, including attachment and internalization, and directly inactivated the virus. Finally, it was found that punicalagin could modulate the NF-κB/STAT3/NLRP3 signaling pathway, reducing ASFV-induced inflammation. These results provide an experimental basis for the use of punicalagin in developing anti-ASFV candidate drugs.

**Abstract:**

African swine fever (ASF), caused by the African swine fever virus (ASFV), has resulted in significant losses in the global pig industry. Considering the absence of effective vaccines, developing drugs against ASFV may be a crucial strategy for its prevention and control in the future. In this study, punicalagin, a polyphenolic substance extracted from pomegranate peel, was found to significantly inhibit ASFV replication in MA-104, PK-15, WSL, and 3D4/21 cells by screening an antiviral compound library containing 536 compounds. Time-of-addition studies demonstrated that punicalagin acted on early viral replication stages, impinging on viral attachment and internalization. Meanwhile, punicalagin could directly inactivate the virus according to virucidal assay. RT-qPCR and Western blot results indicated that punicalagin modulated the NF-κB/STAT3/NLRP3 inflammasome signaling pathway and reduced the levels of inflammatory mediators induced by ASFV. In conclusion, this study reveals the anti-ASFV activity of punicalagin and the mechanism of action, which may have great potential for developing effective drugs against ASFV.

## 1. Introduction

ASF is an acute, febrile, highly contagious hemorrhagic disease caused by the ASFV. ASFV can induce a mortality rate of up to 100% in both domestic pigs and wild boars, which is a severe threat to the global pig industry and causes substantial economic losses [1]. ASFV is a giant double-stranded DNA virus belonging to the *Asfarviridae* family, with a genomic length varying from about 170 to 193 kb due to the mutation of the multi-gene family (MGF) [2]. Since its introduction in China in 2018, ASF has caused significant challenges and severe impacts on the Chinese pig industry [3]. The genomic complexity and variability of ASFV and the complexity of synergistic interactions among multiple ASFV proteins are poorly understood, making it difficult to identify key targets for vaccine development [4]. Additionally, there is low cross-protection between various strains, complicating the screening of effective vaccine candidates, and there is only one approved vaccine at present [5,6]. Therefore, screening and developing antiviral drugs against ASFV is of great significance for clinical prevention and therapy.

Currently, various compound libraries are being utilized to identify potential anti-ASFV drugs. For example, triapine and cytarabine hydrochloride, screened from a library of FDA-approved drugs, have demonstrated dose-dependent inhibition of ASFV replication [7]. Brincidofovir, identified from a kinase inhibitor library, showed significant inhibition of ASFV both in vitro and in vivo [8]. In addition, some natural products have been screened to exert anti-ASFV effects by modulating inflammation and autophagy signaling pathways, such as dihydromyricetin and kaempferol [9,10]. Despite these advancements, no drug has yet been approved for clinical use against ASFV, so more studies are needed to screen effective candidate compounds.

The antiviral compound library (HY-L027) is an extensive commercial resource designed to inhibit a wide range of viruses, making it a valuable asset in the development of antiviral drugs. This library was utilized to screen for compounds with anti-ASFV activity, and punicalagin was identified as a notably effective agent. Punicalagin, an ellagitannin derived from the peel of the pomegranate (*Punica granatum* L.), is known for its multifaceted biological activities, including antioxidant, anti-inflammatory, anti-cancer, antimicrobial, and antiviral effects [11,12,13,14,15]. Subsequently, experiments were designed to verify and explore the mechanisms underlying punicalagin’s anti-ASFV activity, aiming to provide insights into its potential therapeutic applications.

## 2. Materials and Methods

### 2.1. Cells, Virus, and Regents

African green monkey kidney epithelial cells MA-104 and porcine kidney cells PK-15 stored in our laboratory were maintained in Dulbecco’s modified Eagle medium (DMEM) (Thermo Fisher Scientific, Waltham, MA, USA) supplemented with 10% fetal bovine serum (FBS) (Thermo Fisher Scientific, MA, USA) [16]. Wild boar lung cells WSL-R4 (supported by Professor Jun Han, China Agricultural University) and the immortalized pulmonary alveolar macrophages iPAMs (3D4/21, supported by Professor Jianzhong Zhu, Yangzhou University) were cultured in RPMI 1640 medium supplemented with 10% FBS. All the cells were cultured at 37 °C with 5% CO_2_. The ASFV strain GZ201801 (GenBank accession number: MT496893.1), in which the open reading frame of MGF100-1R was replaced by an eGFP expression cassette (referred to as ASFVGZΔMGF100-1R), was prepared in our previous study [17] and stored at −80 °C before use.

The antiviral compound library (HY-L027) and punicalagin (HY-N0063) were purchased from MedChemExpress (Monmouth Junction, NJ, USA). These following antibodies were used in this study: anti-p30, p72 proteins (prepared in our laboratory) [18], anti-β-actin (ab6267, Abcam, Cambridge, UK), anti-STAT3 (SY34-01, Huabio, Hangzhou, China), anti-pSTAT3 (SZ43-01, Huabio, Hangzhou, China), anti-p65 (AN365, Beyotime Biotechnology, Shanghai, China), anti-p-p65 (3033, CST, Danvers, MA, USA), anti-NLRP3 (19771-1-AP, Proteintech, Wuhan, China), anti-caspase-1 (ET1608-69, Huabio, Hangzhou, China), anti-GSDMD (WL05686, Wanleibio, Shenyang, China), anti-IL-1β (A16288, ABclonal, Wuhan, China), and horseradish peroxidase (HRP)-conjugated anti-rabbit and anti-mouse secondary antibody were obtained from Jackson ImmunoResearch Labs (West Grove, PA, USA).

### 2.2. Cell Viability Assay

The CCK-8 assay was used to determine cell viability. Briefly, MA-104, PK-15, 3D4/21, or WSL cells were treated with serial dilutions (10, 20, 40, 80, and 100 μM) of the compound and incubated at 37 °C for 48 h. The cell culture medium was set as the negative control. Subsequently, these cells were incubated with 10 μL-CCK-8 (Vazyme, Nanjing, China) at 37 °C for 1.5 h. The absorbance at 450 nm was measured using a microplate reader (Biotek, Winooski, VT, USA).

### 2.3. Screening of the Antiviral Activity of the Compound Library

The compounds in the antiviral compound library were stored as 10 mM stock solutions in DMSO at −80 °C until use. For the primary screening, MA-104 cells (2 × 10^4^ cells/well) were seeded in 96-well cell plates and incubated overnight. Cells were treated with 10 μM of each compound or DMSO (0.1% *v*/*v*) for 1 h, then infected with 1 TCID_50_ (50% tissue culture infectious dose)/cell ASFVGZΔMGF100-1R for 1 h. Subsequently, the cells were washed with PBS, and a fresh medium containing the corresponding compound (10 μM) was added. At 72 h post-infection (hpi), fluorescence and bright-field images were taken. Image J software (Image J 1.51h, Java 1.6 0_24 64 bit) was used to calculate the fluorescence intensity to determine the inhibitory rate of the compounds, where inhibitory rate = 1—fluorescence intensity of compound-treated cells/fluorescence intensity of DMSO-treated cells. Compounds with inhibitory rates less than 80% and any visible cytotoxicity were excluded. Cell viability of the candidate compounds was further measured at 10 μM.

### 2.4. Anti-ASFV Activity Assay

To further evaluate the anti-ASFV activity of punicalagin, MA-104 cells were treated with different concentrations of punicalagin (ranging from 0 to 20 μM) for 1 h. Subsequently, the cells were infected with 1 TCID_50_/cell ASFVGZΔMGF100-1R for 1 h. The solution was then removed, and the cells were washed three times. Then, the cells were cultured with the corresponding concentration of punicalagin. At 24 hpi, the treated cells were collected for Western blot analysis, and supernatant samples were used to determine the viral gene copy number to calculate the inhibitory rate. The 50% cytotoxic concentration (CC_50_) and 90% inhibitory concentration (IC_90_) were calculated using GraphPad Prism 8.0 software. The same procedure was applied to PK-15, WSL, and 3D4/21 cells to confirm the anti-ASFV activity of punicalagin across various porcine cell lines. At 24 hpi, the treated cells were harvested for RT-qPCR analysis. Furthermore, we investigated the potential of punicalagin to inhibit ASFV replication over an extended period, and the viral gene copies were measured following treatment with 20 μM punicalagin for 24, 48, and 72 h.

### 2.5. Time-of-Addition Assay

For the time-of-addition assay, MA-104 cells (2 × 10^5^ cells/well) cultured in a 24-well cell plate were treated with punicalagin at different hpi. For pre-infection, cells were treated with 20 μM punicalagin for 2 h before ASFVGZΔMGF100-1R (1 TCID_50_/cell) infection (−2 hpi), then punicalagin was removed. For co-infection, 20 μM punicalagin and ASFVGZΔMGF100-1R (1 TCID_50_/cell) were added to the MA-104 cells simultaneously (0 hpi). For post-infection, cells were infected with ASFVGZΔMGF100-1R (1 TCID_50_/cell), and punicalagin was added at 2, 4, 8, 12, or 16 hpi. All cell samples were collected for Western blot analysis, and the supernatant samples were collected for viral gene copy number determination at 24 hpi.

### 2.6. Virucidal Assay

The ASFVGZΔMGF100-1R (1 TCID_50_/cell) was incubated with 20 μΜ punicalagin at 37 °C for 1 h and 3 h. Then, the treated virus suspension was diluted 20-fold to infect MA-104 cells. At 24 hpi, cell samples were harvested for Western blot analysis, and supernatant samples were collected for viral gene copy number determination.

### 2.7. Virus Entry Assay

For the viral attachment assay, MA-104 cells were treated with ASFVGZΔMGF100-1R (1 TCID_50_/cell) and punicalagin (20 μM) and then incubated at 4 °C for 1 h. Subsequently, the cells were washed with PBS to remove any unbound viruses, and a fresh medium with 1% FBS was added. At 24 hpi, cell samples were collected for Western blot analysis, and supernatant samples were collected to determine the viral gene copy number.

For the viral internalization assay, MA-104 cells were infected with 1 TCID_50_/cell ASFVGZΔMGF100-1R at 4 °C for 1 h. Then, the supernatant was removed, replaced with punicalagin (20 μM), and incubated at 37 °C for 1 h, allowing virus entry into the cells. Subsequently, the cells were washed with PBS to remove the compound and replaced with fresh culture medium containing 1% FBS. At 24 hpi, cell samples were collected for Western blot analysis, and supernatant samples were collected to determine the viral copy number.

### 2.8. Fluorescence Imaging

The treated cells were fixed with 4% paraformaldehyde (PFA) at room temperature for 15 min and then washed thrice with PBS. Images were captured using an Olympus IX50 inverted microscope, and the fluorescence intensity was measured with ImageJ software [19].

### 2.9. Western Blotting

The cells grown in a 12-well plate were lysed using RIPA lysis buffer (New Cell & Molecular Biotech, Suzhou, China). Protein quantification was performed using the BCA Protein Quantification Kit (Vazyme, Nanjing, China). Protein samples were separated using SDS-PAGE with 8% or 12.5% gel concentration. After transferring the proteins, the nitrocellulose membrane was blocked with 5% BSA at room temperature for 1 h. The membrane was then incubated with primary antibodies overnight at 4 °C. Following this, it was exposed to HRP-conjugated anti-mouse or anti-rabbit secondary antibodies at room temperature for 1 h. Imaging was performed by the Tanon-5200 Multi-Infrared Imaging System (Tanon Science & Technology Co., Ltd. Shanghai, China).

### 2.10. qPCR and RT-qPCR

The FastPure Cell/Tissue DNA Isolation Mini Kit was used to extract DNA from the supernatant of treated MA-104 cells. Viral gene copies in the cell supernatant were quantified using the absolute quantification method established in our previous study [20]. Following the manufacturer’s instructions, the total RNA from MA-104, PK-15, and WSL cells was extracted using the FastPure Cell/Tissue Total RNA Isolation Kit V2 (Vazyme, Nanjing, China). The extracted RNA was reverse transcribed into cDNA using the HiScript III RT SuperMix (Vazyme, Nanjing, China). RT-qPCR was conducted using SYBR qPCR Master Mix (Vazyme, Nanjing, China) and the LightCycler system (Roche Diagnostics, Mannheim, Germany). The relative quantification of the target gene was determined using the 2^−ΔΔCt^ method, with β-actin serving as the housekeeping gene. Three independent replicates were performed. The primer sequences utilized in this study are listed in Table 1.

### 2.11. Statistical Analysis

The data were expressed as the mean ± standard deviation from three independent experiments. Statistical analysis was conducted using *t*-tests or one-way analysis of variance (ANOVA) with GraphPad Prism 8.0 software (GraphPad Software, USA). In the figures, asterisks indicated significant differences (* *p* < 0.05; ** *p* < 0.01; *** *p* < 0.001), while “ns” indicated not significant.

## 3. Results

### 3.1. Screening of Compounds against ASFV

**Figure 1:**
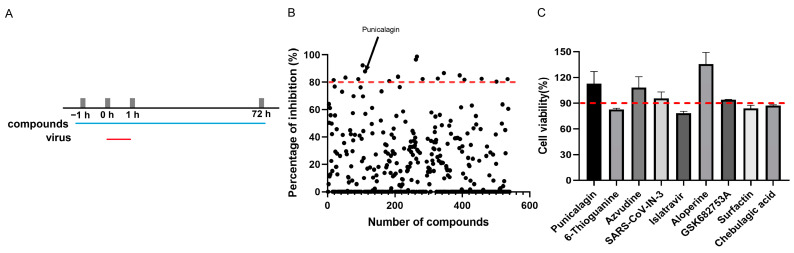
Screening for compounds against ASFV. (**A**) Screening workflow. MA-104 cells were treated with each compound (10 μM) for 1 h, then infected with ASFVGZΔMGF100-1R (1 TCID_50_/cell) for 1 h. Then, the cells were washed with PBS, and fresh medium containing the corresponding compound (10 μM) was added for another 70 h. (**B**) Each dot represents the inhibitory rate of each compound. The dots above the dotted line indicate 80% or more significant inhibition. (**C**) The cell viability of compounds at 10 μM.

As shown in Figure 1A, MA-104 cells were treated with 10 µM compounds and infected with ASFV. By excluding compounds that exhibited visible cytotoxicity, we preliminarily identified 17 compounds with inhibition rates greater than 80% (Figure 1B). Among these, 8 compounds, including (-)-epicatechin gallate, cytarabine, (-)-epigallocatechin gallate, chloroquine, cidofovir, brequinar, emodin (Frangula emodin), and adefovir had been reported to possess anti-ASFV activity [7,21,22,23,24,25], which also validated the feasibility of our screening model. We measured the cell viability of the other nine compounds at 10 μM. It was found that the cell viability of cells treated with punicalagin, azvudine, SARS-CoV-IN-3, aloperine, and GSK682753A were above 90%, while those treated with 6-thioguanine, islatravir, surfactin, and chebulagic acid were below 90% (Figure 1C). In this study, punicalagin (PUG) was selected for further research.

### 3.2. Punicalagin Could Inhibit ASFV Replication In Vitro

**Figure 2:**
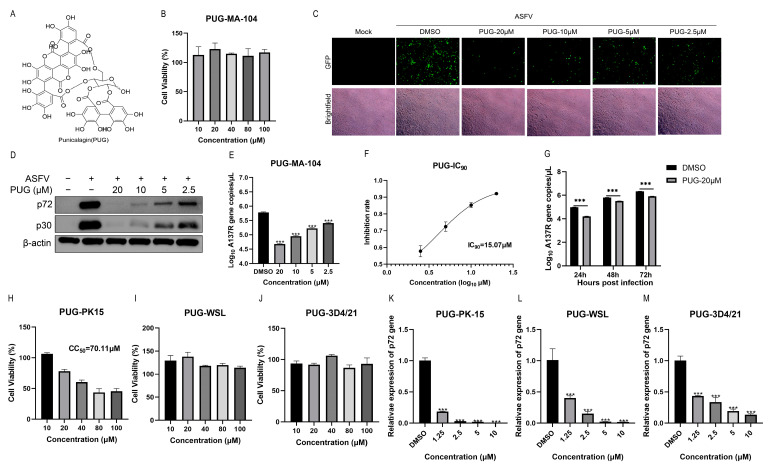
Punicalagin inhibits ASFV replication in vitro. (**A**) Chemical structure of punicalagin. (**B**) The cell viability of punicalagin-treated MA-104 cells. (**C**) The anti-ASFV effect of punicalagin in MA-104 cells was evaluated by fluorescence intensity, scale bar = 100 μm. (**D**,**E**) The anti-ASFV effect of punicalagin in MA-104 cells was determined by Western blot and qPCR. (**F**) The IC_90_ of punicalagin. (**G**) ASFV DNA copies were detected by qPCR after treatment with punicalagin for different time points (24, 48, and 72 h). (**H**–**J**) The cell viability of punicalagin-treated PK-15, WSL, and 3D4/21 cells was detected by CCK-8 assay after treatment for 48 h. (**K**–**M**) The anti-ASFV effect of punicalagin on PK-15, WSL, and 3D4/21 cells was assessed using RT-qPCR, and expression was normalized to GAPDH mRNA. The data were analyzed by GraphPad Prism 8.0. Data were collected from three independent experiments. *** *p*  <  0.001 compared to the respective DMSO control.

We used various assays to systematically evaluate the antiviral efficacy of punicalagin (Figure 2A) against ASFV. First, we used the CCK-8 assay to assess the effects of punicalagin on cell viability in MA-104 cells and found that the CC_50_ of punicalagin was over 100 μM (Figure 2B). Then, MA-104 cells were treated with different concentrations (2.5, 5, 10, and 20 µM) of punicalagin and infected by ASFV. At 24 hpi, the results revealed a significant reduction in fluorescence intensity in the punicalagin-treated groups compared to the DMSO-treated group (Figure 2C). Western blot analysis demonstrated a dose-dependent decrease in ASFV protein expression of p72 and p30 (Figure 2D). The qPCR results also showed a dose-dependent decrease in A137R gene copies, and the viral gene copies reduction was more evident at 20 µM of punicalagin treatment, reducing from 6.79 to 5.68 log_10_ compared to the DMSO-treated group (Figure 2E). The IC_90_ of punicalagin was 15.07 μM calculated by GraphPad Prism 8.0 software (Figure 2F). As shown in Figure 2G, punicalagin could suppress ASFV replication over an extended period by reducing ASFV gene copies (*p* < 0.001) at every time point. To determine the anti-ASFV efficacy of punicalagin in porcine cells, the same experiments were conducted in PK-15, WSL, and 3D4/21 cells. The CCK-8 assay revealed that the CC_50_ of punicalagin in PK-15 cells was 70.11 μM, while WSL and 3D4/21 cells indicated that punicalagin had no cytotoxicity at 100 μM (Figure 2H–J). RT-qPCR results demonstrated that punicalagin reduced the transcription levels of the ASFV p72(*B646L*) gene (*p* < 0.001) dose-dependently in these cells (Figure 2K–M), indicating the antiviral activity of punicalagin against ASFV in various pig cells. These results revealed that punicalagin suppressed ASFV replication dose-dependently in vitro.

### 3.3. Punicalagin Affected the Early Stage of ASFV Infection

**Figure 3:**
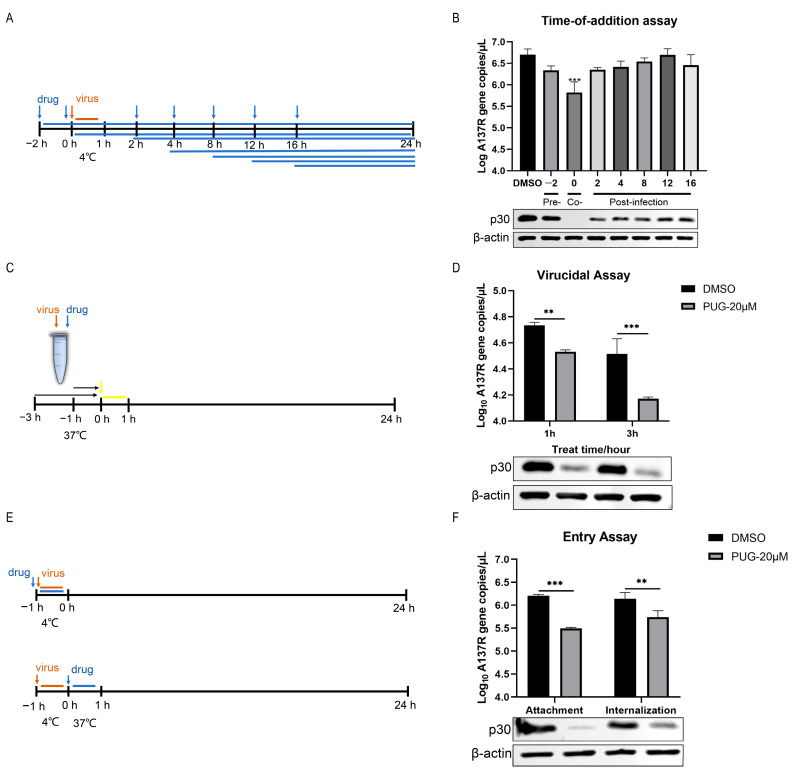
Effect of punicalagin on viral inactivation, entry, and replication stages. (**A**,**B**) Time-of-addition assay. Punicalagin (20 μM) was added at seven different time points: pre-, co-, and post-infection. The anti-ASFV effect was assessed by qPCR and Western blot at 24 hpi, and β-actin was used as a control. (**C**,**D**) Virucidal assay. Punicalagin (20 μM) and ASFVGZΔMGF100-1R (1 TCID_50_/cell) were incubated at 37 °C for 1 h and 3 h. Then, the treated virus suspension was diluted 20-fold to infect MA-104 cells. The anti-ASFV effect was determined by qPCR and Western blot at 24 hpi, and β-actin was used as a control. (**E**,**F**) qPCR and Western blot analysis of the effect of punicalagin on virus entry, including attachment and internalization, and β-actin was used as a control. ** *p*  <  0.01, and *** *p*  <  0.001 compared to the respective DMSO control.

To explore the inhibitory mechanism of punicalagin on ASFV replication, we conducted a time-of-addition experiment (Figure 3A). Punicalagin was added at different time points pre-, co-, and post-infection. The inhibitory impact was measured at 24 hpi. The results showed that the most effective antiviral activity was observed when punicalagin was added immediately after infection (0 hpi), reducing the viral gene copies by about 0.88 log_10_ and p30 protein expression (Figure 3B). Due to the impact of punicalagin on the early stages of ASFV infection, we further investigated its effect on viral inactivation (Figure 3C) and entry stages (Figure 3E). The results showed that pretreating ASFV with punicalagin for 1 h and 3 h reduced the viral gene copy numbers by about 0.2 log_10_ and 0.34 log_10_, respectively, along with the inhibition of p30 protein expression (Figure 3D), which indicated that punicalagin directly inactivated the virus, and this inactivation effect intensified with prolonged treatment time. Furthermore, we found that punicalagin influenced viral attachment and internalization, leading to viral gene copy numbers’ reduction in 0.71 log_10_ and 0.4 log_10_, corroborated by p30 protein expression inhibition (Figure 3F). These results indicated that punicalagin affected viral entry and directly interacted with the virus.

### 3.4. Punicalaginsuppressed the ASFV-Induced NF-κB/STAT3 Pathway

**Figure 4:**
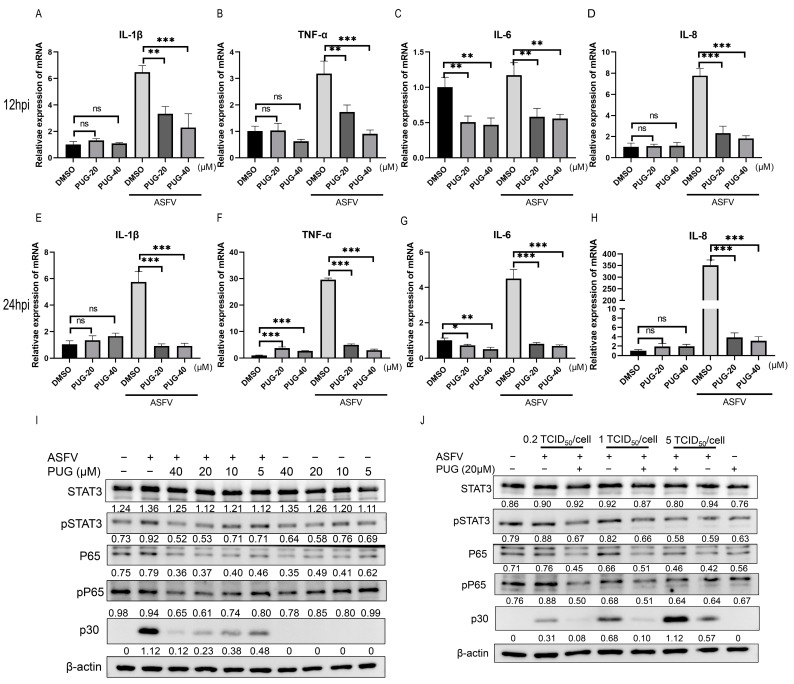
Punicalagin suppressed the ASFV-induced NF-κB/STAT3 pathway. RT-qPCR of ASFV-infected MA-104 cells treated with different concentrations of punicalagin (20 and 40 μM), expression was normalized to β-actin mRNA; samples were collected at 12 h post-infection (hpi) (**A**–**D**) and at 24 hpi (**E**–**H**). (**I**) Western blotting of ASFV-infected MA-104 cells treated with different concentrations of punicalagin (5, 10, 20, and 40 μM), and β-actin was used as a control; samples were collected at 24 hpi. (**J**) Western blotting of MA-104 cells infected with 0.2, 1, or 5 TCID_50_/cell ASFV treated with 20 μM punicalagin, and β-actin was used as a control; samples were collected at 24 hpi. The gray values of p-STAT3, STAT3, p-P65, P65, and p30/actin were shown below the corresponding bands in Western blotting. * *p*  <  0.05, ** *p*  <  0.01, and *** *p*  <  0.001 compared to the respective DMSO control.

Punicalagin has been reported to alleviate chronic and acute inflammation, involving inflammation-related signaling pathways such as NF-κB and STAT3 [26,27]. Here, the mRNA expression levels of IL-1β, TNF-α, IL-6, and IL-8 induced by ASFV were examined after punicalagin treatment at 12 and 24 hpi. The results indicated that ASFV infection significantly activated the transcription of these inflammatory cytokines, while punicalagin treatment dose-dependently reduced the expression of these cytokines (Figure 4A–H). Western blot results revealed that the ASFV-induced phosphorylated STAT3 and p65 expression levels were decreased by punicalagin treatment. (Figure 4I). Moreover, punicalagin treatment reduced these protein expression levels with increasing doses of ASFV (Figure 4J). These results indicated that punicalagin could reduce ASFV-induced inflammatory cytokines via the NF-κB/STAT3 signaling pathway, which could explain ASFV replication decrease.

### 3.5. Punicalagin Inhibited NLRP3 Inflammasome Signaling

**Figure 5:**
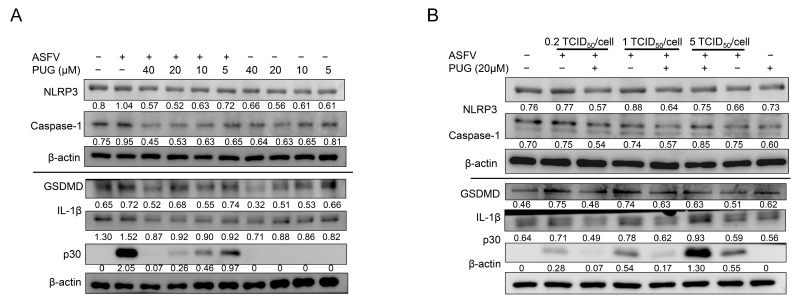
Punicalagin inhibited NLRP3 inflammasome signaling induced by ASFV. (**A**) Western blotting of ASFV-infected MA-104 cells treated with different concentrations of punicalagin (5, 10, 20, and 40 μM) and β-actin was used as a control; samples were collected at 24 hpi. (**B**) Western blotting of MA-104 cells infected with 0.2, 1, or 5 TCID_50_/cell ASFV treated with 20 μM punicalagin and β-actin was used as a control; samples were collected at 24 hpi. The gray values of NLRP3, caspase-1, GSDMD, IL-1β, and p30/actin were shown below the corresponding bands in Western blotting.

The formation of NLRP3 inflammasome is associated with the activation of NF-κB/STAT3 signaling pathway. It has been reported that ASFV infection could induce IL-1β production via activating NLRP3 inflammasome and increased LDH secretion, suggesting that ASFV could induce pyroptosis in primary porcine pulmonary alveolar macrophages (PAMs) [28,29]. Here, the protein expression levels of NLRP3 and caspase-1 were investigated. The Western blot results showed that ASFV infection promoted their expression, but treatment with punicalagin inhibited their expression dose-dependently under different multiplicities of infection (MOIs). In addition, ASFV infection increased the protein expression of IL-1β and GSDMD, but treatment with punicalagin to different MOIs could reduce their expression (Figure 5A,B). These results suggested that punicalagin treatment could inhibit the NLRP3 inflammasome signaling pathway induced by ASFV.

## 4. Discussion

As an ancient virus, ASFV was first discovered in Kenya, Africa, in 1921 [27] and has a history of over 100 years. Despite extensive efforts, there is only one approved vaccine available for ASFV in Vietnam, although its long-term efficacy and safety have not been established. As a result, there is an urgent need for the discovery and development of novel therapeutic agents against this virus. MA-104 cells have been reported to be susceptible to ASFV infection in vitro, making them a suitable model for studying ASFV infection-related biological processes [16,30,31,32]. We compared ASFV replication across various cell lines and observed that ASFV exhibited significantly better growth in MA-104 cells compared to swine cell lines, including PK-15, WSL, and 3D4/21 cells [33]. So, we chose MA-104 cells for our research. Notably, these cell lines have specific mutations that make them very permissive for many viruses, since their innate defense mechanisms are impaired. It would be better if we use PAMs, the primary target cells of ASFV. In this study, we screened an antiviral compound library and found that punicalagin significantly inhibited ASFV replication in vitro. Further investigations revealed that punicalagin primarily acts during the entry phase of ASFV infection and can directly inactivate the virus (Figure 3B,D,F).

Punicalagin has been reported to exhibit antiviral activity against various viruses that utilize glycosaminoglycans (GAGs) for cell entry, manifesting both viral inactivation and inhibition of viral entry [13,14], akin to the findings of our current ASFV research. Heparin, known for interacting with cell surface glycosaminoglycan-heparan sulfate (HS), effectively blocks the entry of HSV-1 and SARS-CoV-2 into cells by competing with HS [34,35]. However, heparin has been shown to have a minimal inhibitory effect on ASFV [36]. Consequently, punicalagin is unlikely to inhibit ASFV entry through competition with GAGs. Further study is needed to investigate the specific interactions between punicalagin and ASFV.

As reported, ASFV infection can trigger a systemic cytokine storm, resulting in the overexpression of pro-inflammatory cytokines [37]. Gao et al. utilized transcriptomics and proteomics to investigate ASFV-infected PAMs, and revealed that ASFV infection activated the NF-κB signaling pathway, leading to increased expression of IL-1β and IL-8, while suppression of NF-κB inhibited ASFV replication [38]. Another study noted that early-stage ASFV infection upregulated IL-6, which activated the JAK2-STAT3 pathway and promoted viral replication [39]. In this study, we confirmed that ASFV significantly upregulated the mRNA expression of IL-1β, TNF-α, IL-6, and IL-8 in MA-104 cells. However, punicalagin treatment effectively reduced these inflammatory cytokines’ expression and attenuated ASFV-induced phosphorylation of p65 and STAT3. Remarkably, punicalagin was the first compound reported to inhibit pyroptosis execution and IL-1β release [40]. In this study, cells treated with punicalagin effectively reduced the expression of NLRP3 and caspase-1 induced by ASFV, along with the expression of GSDMD and IL-1β. In a recent study, Jackman et al. screened a library of natural products with anti-inflammatory activity and found that berbamine and tetrandrine significantly inhibited ASFV replication by affecting viral entry [41]. Additionally, they markedly suppressed the production of inflammatory cytokines induced by ASFV, which is similar to our findings with punicalagin. Overall, these findings reveal that punicalagin may exert its anti-ASFV effects by modulating the NF-κB/STAT3/NLRP3 inflammasome signaling pathway and suppressing the production of inflammatory factors.

In this study, we found that punicalagin has a unique mechanism of action targeting viral entry and directly interacts with the virus, which differs from the mechanisms of triapine (a ribonucleotide reductase inhibitor) and cytarabine hydrochloride (a nucleoside analog). In addition, punicalagin could exert anti-inflammatory effects, which may be helpful in inhibiting a cytokine storm caused by ASFV. As a natural product, punicalagin may have fewer side effects and a more favorable toxicity profile compared to brincidofovir. Punicalagin’s distinct mechanism and natural origin could complement existing antiviral agents like brincidofovir in combination therapies, potentially enhancing overall efficacy and reducing resistance.

Although preclinical studies have demonstrated promising therapeutic effects of punicalagin [42], its clinical application is substantially limited by poor bioavailability [43]. Molecular reduction and encapsulation strategies are employed to enhance the bioavailability of punicalagin. For instance, when encapsulated in capsule form, pomegranate extract contains high levels of punicalagin and is marketed as a nutraceutical to support cardiovascular health [44]. However, to evaluate the therapeutic potential of punicalagin, especially in the context of viral infections such as ASFV, further research is essential to determine its optimal therapeutic dose in vivo. This will involve exploring various formulations and delivery methods to improve its systemic availability and therapeutic efficacy.

## 5. Conclusions

Punicalagin was found to exert a dose-dependent inhibitory effect on ASFV in MA-104, PK-15, and WSL cells. It was demonstrated that punicalagin acted on the entry stage of viral infection and could directly interact with ASFV to inactivate the virus. Moreover, punicalagin modulated the NF-κB/STAT3/NLRP3 inflammasome signaling pathway to suppress viral replication. These findings provide a possible drug candidate against ASFV.

## Figures and Tables

**Table 1:** Primers utilized in this study.

Target	Sequence (5′–3′)
ASFV-A137R-F	GGACATCGAGTGGTATTAAAAGG
ASFV-A137R-R	TGGCCTGAAAGTCAACATTGA
β-actin (monkey)-F	TCGATCATGAAGTGCGACGTG
β-actin (monkey)-R	GTGATCTCCTTCTGCATCCTGTC
IL-1β (monkey)-F	TAGACCTCTGCCCTCTGGAT
IL-1β (monkey)-R	CTCCATGGCTACAACAACCG
TNF-α (monkey)-F	CTGCACTTTGGAGTGATCGG
TNF-α (monkey)-R	GCTACAGGCTTGTCACTTGG
IL-6 (monkey)-F	GGAACGAAAGAGAAGCTCTA
IL-6 (monkey)-R	CTTGTGGAGACGGAGTTCA
IL-8 (monkey)-F	AGCTCTGTGTGAAGGTGCAG
IL-8 (monkey)-R	CAGAGCTCTCTTCCATCAGAAA
GAPDH (pig)-F	CAAGGCTGTGGGCAAGGTCATC
GAPDH (pig)-R	CACGAGGAAGCAAGCAGAGTCAG
ASFV-p72-F	CTGCTCATGGTATCAATCTTATCGA
ASFV-p72-R	GATACCACAAGATCAGCCGT

## Data Availability

The data supporting this study’s findings are available from the corresponding author (A.Q.) upon reasonable request.
